# Transcriptomic comparison of bone marrow CD34 + cells and peripheral blood neutrophils from ET patients with *JAK2* or *CALR* mutations

**DOI:** 10.1186/s12863-023-01142-5

**Published:** 2023-08-07

**Authors:** Ana Guijarro-Hernández, José Luis Vizmanos

**Affiliations:** Department of Biochemistry and Genetics, School of Sciences, University of Navarra, Pamplona, Spain

**Keywords:** Myeloproliferative neoplasms, Essential thrombocythemia, Bone marrow, Peripheral blood, JAK2, CALR

## Abstract

**Background:**

Essential thrombocythemia (ET) is one of the most common types of *Ph*-negative myeloproliferative neoplasms, an infrequent group of blood cancers that arise from a CD34 + hematopoietic stem cell (HSC) in the bone marrow (BM) primarily due to driver mutations in *JAK2*, *CALR* or *MPL*. These aberrations result in an overproduction of mature myeloid cells in peripheral blood (PB). To date, no targeted therapies have been approved for ET patients, so the study of the molecular mechanisms behind the disease and the identification of new therapeutic targets may be of interest. For this reason, in this study, we have compared the transcriptomic profile of undifferentiated CD34 + cells and mature myeloid cells from ET patients (*CALR* and *JAK2*-mutated) and healthy donors deposited in publicly available databases. The study of the similarities and differences between these samples might help to better understand the molecular mechanisms behind the disease according to the degree of maturation of the malignant clone and the type of mutation and ultimately help identify new therapeutic targets for these patients.

**Results:**

The results show that most of the altered hallmarks in neutrophils were also found in CD34 + cells. However, only a few genes showed a similar aberrant expression pattern in both types of cells. We have identified a signature of six genes common to patients with *CALR* and *JAK2* mutations (*BPI*, *CRISP3*, *LTF*, *MMP8*, and *PTGS1* upregulated, and *PBXIP1* downregulated), a different signature of seven genes for patients with *CALR* mutations (*BMP6*, *CEACAM8*, *ITK*, *LCN2*, and *PRG2* upregulated, and *MAN1A1* and *MME* downregulated) and a signature of 13 genes for patients with *JAK2* mutations (*ARG1*, *CAST*, *CD177*, *CLEC5A*, *DAPP1*, *EPS15*, *IL18RAP*, *OLFM4*, *OLR1*, *RIOK3*, *SELP*, and *THBS1* upregulated, and *IGHM* downregulated).

**Conclusions:**

Our results highlight transcriptomic similarities and differences in ET patients according to the degree of maturation of the malignant clone and the type of mutation. The genes and processes altered in both CD34 + cells and mature neutrophils may reveal altered sustained processes that could be studied as future therapeutic targets for ET patients.

**Supplementary Information:**

The online version contains supplementary material available at 10.1186/s12863-023-01142-5.

## Background

*Ph*-negative myeloproliferative neoplasms (MPNs, ET: essential thrombocythemia; PV: polycythemia vera; PMF: primary myelofibrosis) are an infrequent group of blood cancers which arise from a CD34 + hematopoietic stem cell (HSC) in the bone marrow mainly due to driver mutations in *JAK2*, *CALR* or *MPL*. These alterations induce the transformation of normal cells into malignant ones, finally causing an overproduction of mature myeloid cells through multiple molecular mechanisms [1, 2]. The main pathogenic mechanism seems to be the constitutive activation of JAK2-related pathways (JAK2/STAT, MAPK/ERK, and PI3K/AKT), but some other non-canonical mechanisms derived from mutant JAK2 and CALR have also been described [1]. However, there are still several issues that have not been fully resolved, such as the fact that the same alterations can lead to different, although related, clinical phenotypes.

ET is one of the most common types of *Ph*-negative MPNs, with an incidence of 1.2-3.0/100,000 per year [3]. This disease is characterized by excessive production of megakaryocytes in the bone marrow, which results in a high platelet count in the peripheral blood (thrombocytosis). According to a recent Mayo Clinic study, ET patients have a significantly shorter survival rate than that of age- and sex-matched controls from the general population. In some cases, this occurs because they show leukemic or fibrotic transformation [4].

At this time, the only targeted therapy approved for some *Ph*-negative MPN patients is ruxolitinib, a JAK1/JAK2 inhibitor. This molecule has been approved for patients with intermediate or high-risk MF and with PV resistant or intolerant to chemotherapy [5]. However, clinical trials suggest that ruxolitinib is not superior to current second line treatments for ET [6]. In this sense, the study of the molecular mechanisms behind the disease and the identification of new potential therapeutic targets for ET patients may be of special interest.

For this reason, in this work we will compare the transcriptomic profile of bone marrow (BM) CD34 + cells and peripheral blood (PB) neutrophils from ET patients and healthy donors deposited in publicly available databases. The comparison of the differentially expressed genes in these BM and PB *JAK2* and *CALR*-mutated ET samples compared to healthy donors might help to better understand the altered processes and molecular mechanisms underlying this disease according to the degree of maturation of the malignant cell (immature CD34 + HSCs from BM vs. mature neutrophils from PB) and the type of mutation. Furthermore, the identification of equally altered hallmarks and similarly aberrantly expressed genes in both types of cells of ET patients could provide clues about the conserved processes in mature neutrophils, which may be quantitatively o functionally important in the pathogenesis of the disease for patients harboring *JAK2* or *CALR* mutations and ultimately could help develop new therapeutic strategies.

## Methods

### Data source

**Fig. 1:**
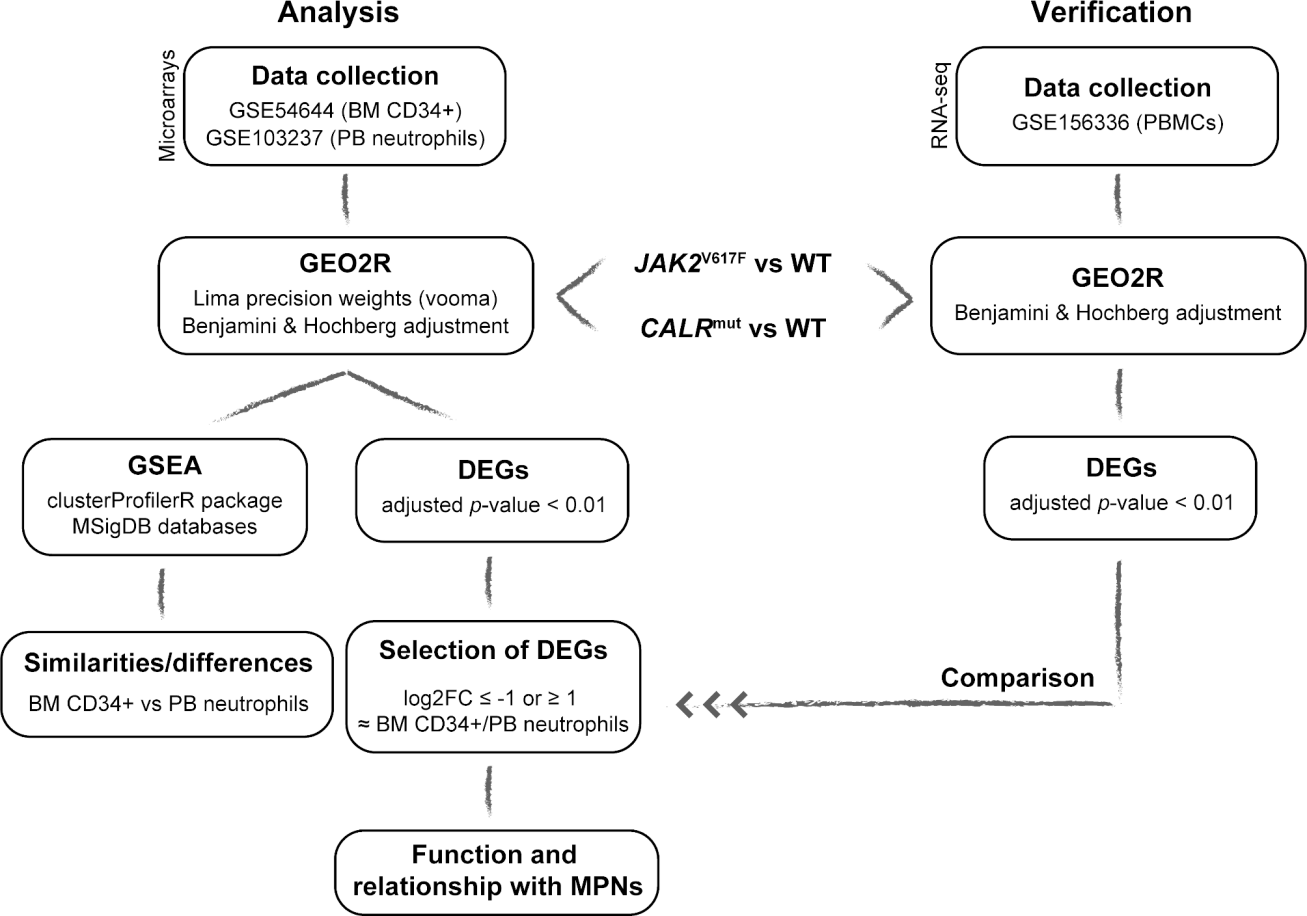
Bioinformatic analysis pipeline. The flowchart summarizes the steps and tools used for building signatures and analyzing the differences between CD34 + cells from BM and PB neutrophils of ET patients, as well as for the validation of the results with RNA-seq data

Transcriptomic data from ET patients (GSE54644, [U133AAofAv2] Affymetrix GeneChip HT-HG_U133A Early Access Array; GSE103237, [HG-U219] Affymetrix Human Genome U219 Array) were obtained from the publicly available Gene Expression Omnibus (GEO) database (www.ncbi.nlm.nih.gov/geo). The GSE54644 set contained expression data from PB neutrophils obtained from 39 ET patients (14 *CALR*-mutated and 25 *JAK2*-mutated) and 11 healthy donors [7]. On the other hand, the GSE103237 dataset consisted of CD34 + cell transcriptomic data from BM obtained from 24 ET patients (7 *CALR*-mutated and 17 *JAK2*-mutated) and 15 healthy donors [8] (Table S1; Fig. 1).

For validation of the obtained results, RNA-seq experiments were analyzed in an independent cohort of patients with ET (3 *CALR*-mutated, 3 *JAK2*-mutated and 4 healthy donors) using peripheral blood mononuclear cell (PBMC) samples (GSE156336, Illumina HiSeq 2500) obtained from the GEO database (Table S1; Fig. 1).

The three datasets used in this study were the only ones found in the GEO database when searching for transcriptomic data of patients with ET with mutations in *CALR* and *JAK2* that also contained data from healthy controls.

### Data processing

The GEO2R tool (https://www.ncbi.nlm.nih.gov/geo/geo2r/) was used to analyze microarray data (GSE54644 and GSE103237) applying lima precision weights (vooma) and the Benjamini & Hochberg adjustment. For each dataset, expression data from *CALR*-mutated and *JAK2*-mutated ET patients were independently compared with data from healthy individuals. All the transcripts with an adjusted *p*-value < 0.01 were considered as differentially expressed genes (DEGs) (Table S2 for *CALR*-mutated vs. healthy donors and Table S3 for *JAK2*-mutated vs. healthy donors). Then, we analyzed the genes that were similarly expressed in both PB and BM samples from *CALR* (Table S4) or *JAK2*-mutated (Table S5) patients selecting those with log2 fold change (log2FC) ≤ -1 or ≥ 1. The expression of these genes was validated by RNA-seq in independent PBMC samples from patients with ET (GSE156336) using the GEO2R tool with Benjamini & Hochberg adjusted *p*-values. Finally, we studied their function and relationship with MPNs (Fig. 1).

Gene Set Enrichment Analysis (GSEA) of gene expression microarray data (GSE54644 and GSE103237) was performed using the clusterProfiler R package [9] on MSigDB databases for humans obtained with the R package msigdbr [10] (Fig. 1).

Graphics were created using GraphPad Prism 8.0.2 (GraphPad Software Inc., San Diego, CA, USA).

## Results

### Main similarities and differences between BM CD34 + cells and PB neutrophils from ***JAK2*** and ***CALR***-mutated ET patients

**Fig. 2:**
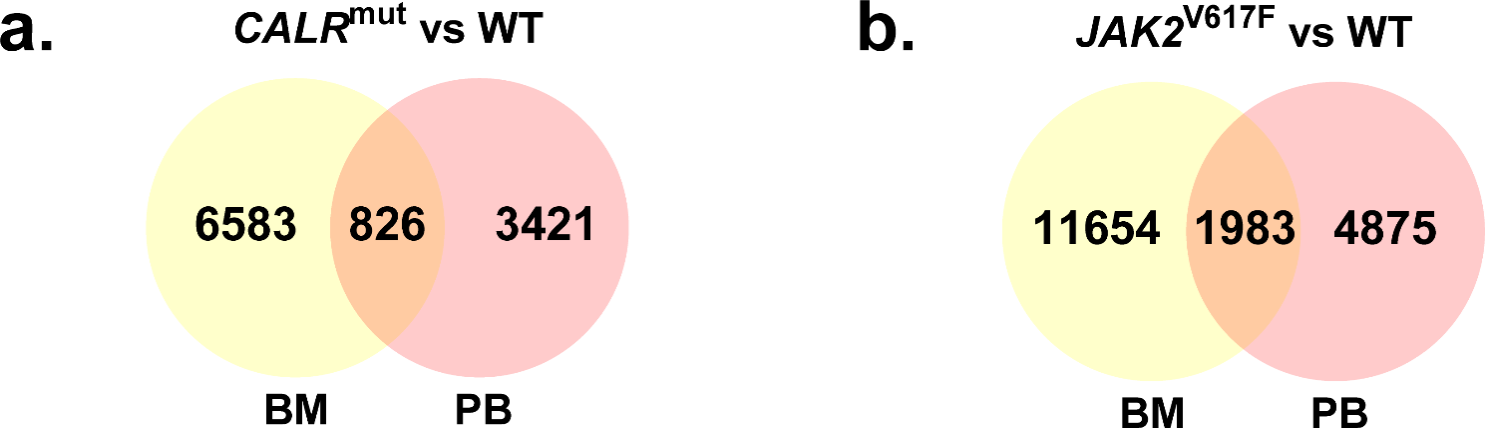
Venn diagrams showing the number of DEGs observed in BM CD34 + cells and PB neutrophils from (**a**) *CALR* and (**b**) *JAK2*-mutated ET patients vs. healthy donors (WT).

Analysis of microarray data revealed that the transcriptomic differences between ET patients and healthy donors are greater in immature CD34 + cells from BM than in mature neutrophils from PB. In fact, the amount of DEGs (Fig. 2; Tables S2, S3) in CD34 + cells was approximately double that in neutrophils for both *CALR* and *JAK2*-mutated patients compared to healthy donors.

In general, patients with *JAK2* mutations showed greater transcriptomic differences when compared to healthy donors than those with *CALR* mutations. Furthermore, we only found 826 common DEGs (338 equally expressed and 488 with opposite expression results) when CD34 + cells and neutrophils data from *CALR*-mutated patients were compared with data from healthy donors (Fig. 2a; Table S4), but there were 1983 common DEGs (919 equally expressed and 1064 with opposite expression results) when comparing data from both cell types in patients with *JAK2* mutations with data from healthy donors (Fig. 2b; Table S5).

### Comparison of the altered processes in BM CD34 + cells and PB neutrophils from ***JAK2*** and ***CALR***-mutated ET patients

**Fig. 3:**
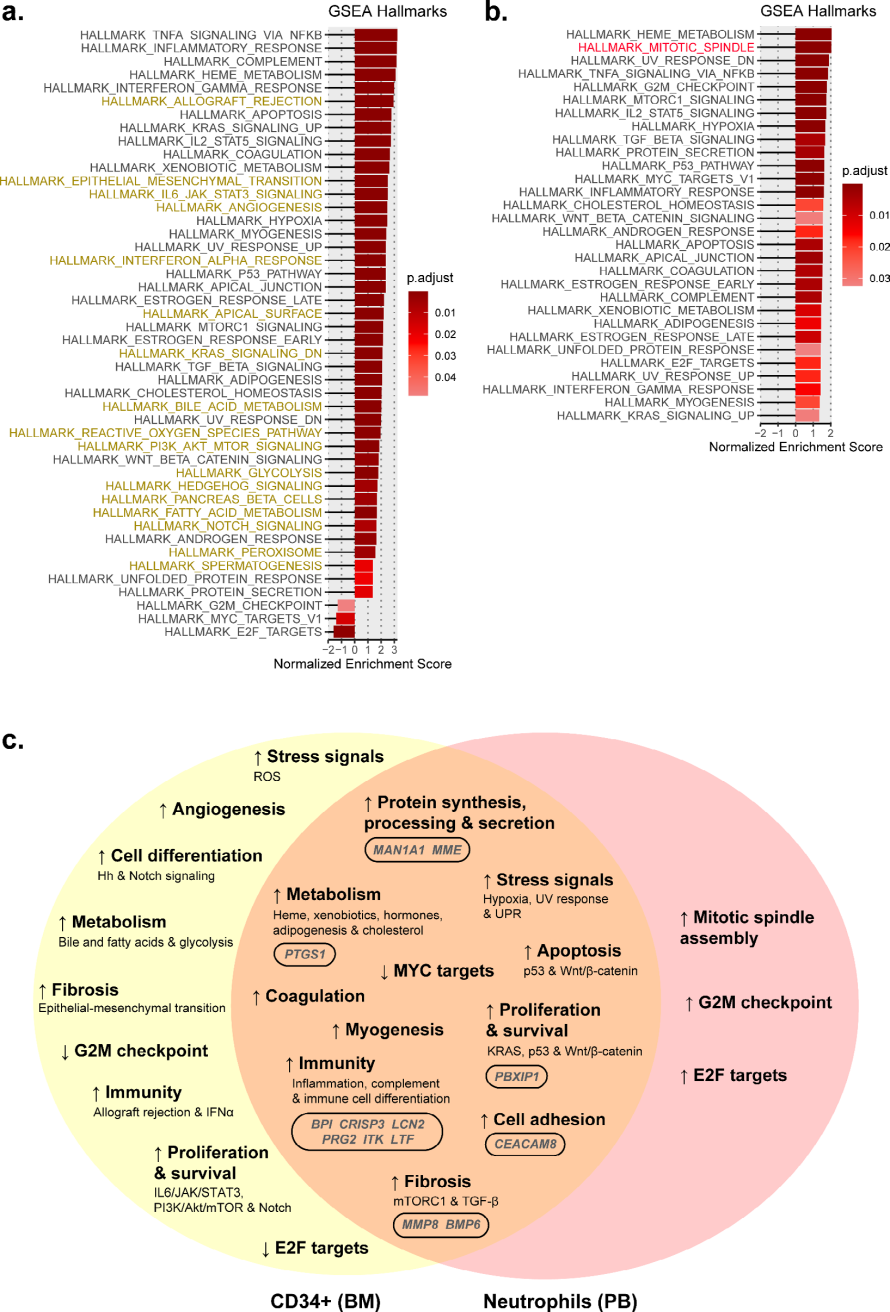
Main altered processes in CD34 + cells from BM and PB neutrophils from ET patients with *CALR* mutations. Hallmarks found in (**a**) CD34 + cells from BM and (**b**) neutrophils from PB samples from ET patients with *CALR* mutations using the GSEA normalized enrichment scores. A darker red color is indicative of a lower GSEA-adjusted *p*-value. The uncolored hallmarks are common to both types of samples while the yellow and red colored hallmarks are exclusive to BM CD34 + cells and PB neutrophils, respectively. **(c)** Venn diagrams that summarize the main altered processes in BM CD34 + cells and PB neutrophils and common gene expression signature for both cell types

**Fig. 4:**
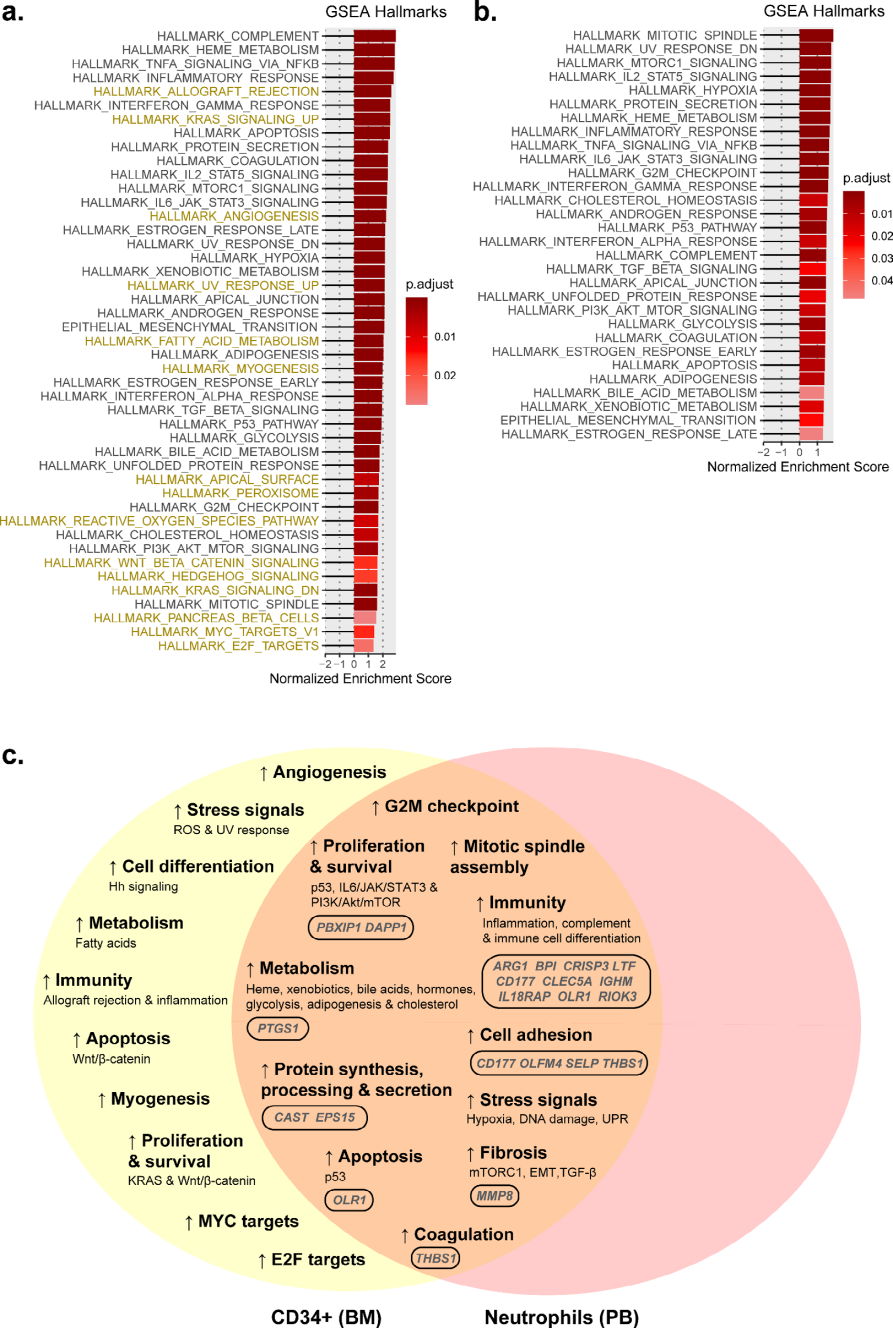
Main altered processes in CD34 + cells from BM and PB neutrophils from ET patients with *JAK2* mutations. Hallmarks found in (**a**) BM CD34 + cells and (**b**) PB neutrophils from ET patients with *JAK2* mutations using the GSEA normalized enrichment scores. A darker red color is indicative of a lower GSEA-adjusted *p*-value. The uncolored hallmarks are common to both types of samples while the yellow and red colored hallmarks are exclusive to BM CD34 + cells and PB neutrophils, respectively. **(c)** Venn diagrams that summarize the main altered processes in BM CD34 + cells and PB neutrophils and common gene expression signature for both cell types

Several hallmarks were exclusively altered in immature CD34 + cells or mature neutrophils. For *CALR*-mutated patients, 17 hallmarks involved in a wide variety of processes were found to be altered exclusively in BM CD34 + cells, but only one hallmark (mitotic spindle assembly) was exclusively altered in PB neutrophils (Fig. 3). For *JAK2*-mutated patients, 16 unique hallmarks were found in BM CD34 + cells, and none were found in PB neutrophils (Fig. 4). Regarding the hallmarks exclusively altered in BM CD34 + cells, it is of interest to note that some of them were shared between patients with *JAK2* and *CALR* mutations (allograft rejection, angiogenesis, apical surface, reactive oxygen species pathway, hedgehog signaling, pancreas beta cells, fatty acid metabolism, and peroxisome), while others were only found in patients with *CALR* mutations (epithelial-to-mesenchymal transition, interferon alpha response, bile acid metabolism, glycolysis, IL6/JAK/STAT3, PI3K/Akt/mTOR and Notch signaling, and spermatogenesis) or *JAK2* mutations (UV response, myogenesis, KRAS signaling, Wnt/β-catenin, and upregulation of MYC and E2F targets). When studying the general biological processes in which these hallmarks were involved, we observed that most of them were commonly altered in patients with mutations in *JAK2* and *CALR*, although the mechanisms by which they are altered may differ. For example, metabolism (biological process) is altered in patients with *JAK2* and *CALR* mutations, but the specific metabolic pathways (hallmarks) altered in patients with each mutation are different. On the other hand, only a few biological processes were exclusively altered in patients with mutations in *CALR* (fibrosis) and *JAK2* (apoptosis and myogenesis).

Some hallmarks were exclusive to BM CD34 + cells or PB neutrophils, but most of the altered ones in PB neutrophils were also found altered in BM CD34 + cells (Figs. 3 and 4). This is not surprising, since MPNs originate in the BM. We focused on them since, being conserved in mature neutrophils (i.e., they remain altered throughout the entire malignant hematopoietic lineage), they might be relevant to the development or progression of the disease. In other words, the biological processes in which they are involved could be important in the pathogenesis of the disease, either quantitatively (due to the significant alteration of one of the hallmarks of the process) or functionally (because the cumulative alteration of multiple hallmarks affects a relevant biological process). Thus, the progression of the disease to MF or the severity of symptoms presented in patients could ultimately depend on these biological processes.

#### Potential hallmarks of interest for ET patients with CALR and JAK2 mutations

Several hallmarks shared between samples from both types of cells were common to both *CALR* and *JAK2*-mutated patients. These were related to activation of stress responses (hypoxia, UV and UPR), immunity (inflammation, interferon gamma response, TNF-α activation via NFκB, complement, and IL2/STAT5 signaling), metabolism (cholesterol homeostasis, adipogenesis, metabolism of heme and xenobiotics, and androgen and estrogen early and late responses), fibrosis (mTORC1 and TGF-β), cell adhesion disruption (apical junction), protein secretion, coagulation, and other processes typically altered in cancer, such as apoptosis (p53 pathway) and proliferation and survival (p53 pathway) (Figs. 3 and 4).

#### Potential hallmarks of interest for ET patients with CALR mutations

Exclusively for patients with *CALR* mutations, the shared hallmarks between BM CD34 + cells and PB neutrophils were involved in the activation of several processes, such as immunity (KRAS signaling), apoptosis (Wnt/β-catenin), proliferation and survival (Wnt/β-catenin and KRAS signaling), and myogenesis. Interestingly, the G2M checkpoint and MYC and E2F targets were enriched in PB neutrophils but repressed in BM CD34 + cells (Fig. 3).

#### Potential hallmarks of interest for ET patients with JAK2 mutations

Hallmarks of interest unique to patients with *JAK2* mutations were associated with the activation of immune responses (interferon alpha response), metabolism (glycolysis and bile acid metabolism), proliferation and survival (IL6/JAK/STAT3 and PI3K/Akt/mTOR pathways), cell adhesion (epithelial-to-mesenchymal transition), mitotic spindle assembly, and the G2M checkpoint (Fig. 4).

### Genes equally expressed in BM CD34 + cells and PB neutrophils from ET patients

**Fig. 5:**
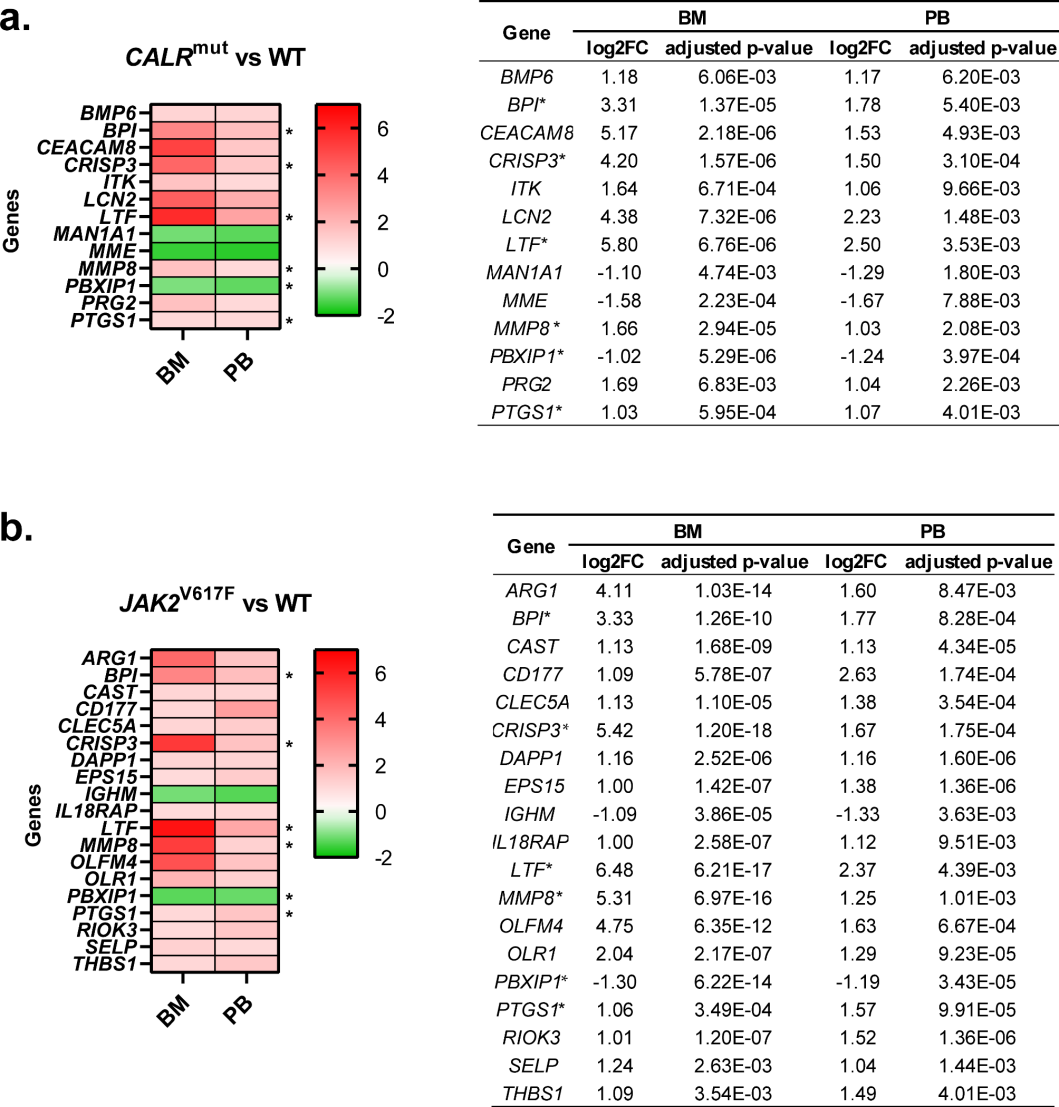
Heatmaps and tables showing data from similarly expressed genes in the BM CD34 + cells and PB neutrophils from patients with (**a**) *CALR* and (**b**) *JAK2* mutations that show a log2FC ≤ -1 or ≥ 1 and an adjusted *p*-value < 0.01 compared with BM and PB samples from healthy controls. Mean fold change values are represented, ranging from shades of red (positive fold change) to shades of green (negative fold change). The common genes found in *CALR* and *JAK2* mutated samples are identified with an asterisk

Analysis of aberrantly and similarly expressed genes (up or downregulated) in BM and PB samples from ET patients and their function is of particular interest because they could reveal altered sustained biological processes that are triggered by *CALR* and *JAK2* mutations. In addition to functional hallmarks, we could detect some similarly expressed genes in BM CD34 + cells and PB neutrophils of patients. These genes participate in a wide variety of processes (Tables S4, S5) and as mentioned above, we focused on the functional analysis of the genes which a log2FC ≤ -1 or ≥ 1 in both types of samples (Figs. 3, 4 and 5).

A total of 13 genes (10 upregulated and 3 downregulated) fulfilled these criteria in samples from patients with *CALR* mutations (Fig. 5a) and 19 genes (17 upregulated and 2 downregulated) in samples from patients harboring *JAK2* mutations (Fig. 5b**)**. Only six of these genes were common to both types of mutations (marked with an asterisk in Fig. 5).

#### Potential targets of interest for ET patients with CALR and JAK2 mutations

All six DEGs that were found in common in samples from patients with both types of mutations (*BPI*, *CRISP3, LTF*, *MMP8*, *PBXIP1*, and *PTGS1*) were upregulated except *PBXIP1*, which was downregulated (Fig. 5**)**. The majority of them participate in immune processes (*BPI*, *CRISP3*, *LTF*), while the remaining ones are related to fibrosis (*MMP8*), proliferation and survival (*PBXIP1*) or metabolic processes involved in the biosynthesis of platelets (*PTGS1*) (Figs. 3 and 4; Table 1). Interestingly, aberrations in all these genes have previously been linked to blood cancers (Table 1).

The study of gene expression in PBMCs from an independent cohort of patients by RNA-seq confirmed that *CRISP3*, *LTF*, *MMP8*, and *PTGS1* were significantly upregulated in patients with mutations in *CALR*, and *PTGS1* in patients with mutations in *JAK2*. The expression of the remaining genes followed a similar trend as observed in BM CD34 + cells and PB neutrophils in the majority of cases, but the results were not statistically significant (Supplementary Figs. 1, 2).

#### Potential targets of interest for ET patients with mutations in CALR

In the case of *CALR*-mutated ET patients, seven genes were aberrantly expressed both in PB and BM samples (*BMP6, CEACAM8, ITK, LCN2, MAN1A1, MME*, and *PRG2*). All of them were upregulated except for *MAN1A1* and *MME*, which were downregulated (Fig. 5a). Most of these genes participate in immune processes (*ITK*, *LCN2*, and *PRG2*) and protein synthesis, processing, and secretion (*MAN1A1* and *MME*). The remaining ones are involved in fibrosis (*BMP6*) and cell adhesion (*CEACAM8*) (Fig. 3; Table 2).

The results obtained by RNA-seq in PBMCs confirmed that *BMP6*, *CEACAM8*, and *LCN2* were upregulated in patients with mutations in *CALR*. No significant results were obtained for the remaining genes (Supplementary Figs. 1, 2).

#### Potential targets of special interest for JAK2-mutated ET patients

In samples from ET patients with *JAK2* mutations, we focused on the analysis of 13 genes (*ARG1, CAST, CD177, CLEC5A, DAPP1, EPS15, IGHM, IL18RAP, OLFM4, OLR1, RIOK3, SELP*, and *THBS1*). All of them, except *IGHM*, were upregulated in both BM and PB-derived samples (Fig. 5b). Most of these genes participate in processes related to immune response (*ARG1*, *CD177*, *CLEC5A*, *IGHM*, *IL18RAP*, *OLR1*, and *RIOK3*) or cell adhesion (*CD177*, *OLFM4*, *SELP*, and *THBS1*), while some of them are involved in processes such as protein synthesis, processing, and secretion (*CAST* and *EPS15*) apoptosis (*OLR1*), proliferation and survival (*DAPP1*), or coagulation (*THBS1*) (Fig. 4; Table 3).

The results obtained in PBMCs confirmed that *OLR1* was upregulated in patients with mutations in *JAK2*. No significant results were obtained for the remaining genes, although the trends were consistent with those observed in BM CD34 + cells and PB neutrophils in many cases (Supplementary Figs. 1, 2).

**Table 1:** Functions and relationship with MPNs of the potential biomarkers of interest for ET patients with *CALR* and *JAK2* mutations

Gene	Protein	Function and relationship with MPNs
***BPI***	Bactericidal permeability increasing protein	Encodes for an antimicrobial lipopolysaccharide binding protein stored in the azurophil granules of neutrophils, in the granules of eosinophils and on the surface of monocytes. BPI-expression during myeloid differentiation can be cooperatively and directly mediated by RUNX1, PU.1, and Sp3 [11]. *RUNX1* mutations are found in hematological malignancies [12] and the overexpression of RUNX1 has an important role in the development of MPNs [13]. The involvement of the RUNX1 pathway in the leukemic transformation of MPNs also seems to be a common event [14]. PU.1 is a key transcription factor required for myeloid differentiation and the p.V617F JAK2 mutation upregulates its expression in the PB of MPN patients [15]. The transcription factor Sp3 is involved in the regulation of many hematopoietic-specific genes [16]. SP family members are also essential for the transcriptional regulation of *CALR* [17].
***CRISP3***	Cysteine-rich secretory protein 8	Encodes for a member of the cysteine-rich secretory protein (CRISP) family within the CRISP, antigen 5 and pathogenesis-related 1 proteins superfamily. Previously described as upregulated as part of a 7-gene predictive signature of early primary prefibrotic myelofibrosis (prePMF) [18]. It has also been identified as a potential PB biomarker for multiple myeloma [19]. Its role in MPNs remains unclear.
***LTF***	Lactotransferrin	Encodes for a member of the transferrin protein family that is also found in the secondary granules of neutrophils acting as a potent inhibitor of granulocyte-macrophage colony stimulating factor production when it binds to monocytes and macrophages [22]. This protein is an important component of the non-specific immune system and a major iron-binding protein in milk and body secretions with an antimicrobial activity and a broad spectrum of properties, including regulation of iron homeostasis, host defense against a broad range of microbial infections, anti-inflammatory activity, regulation of cellular growth and differentiation and protection against cancer development and metastasis. A decrease in the presence [23] and release [24] of lactotransferrin from the neutrophils has been reported in patients with MPNs and may be responsible for an increased myeloid cells proliferation. This gene has been found upregulated in PV, ET and PMF [25].
***MMP8***	Matrix metalloproteinase 8	Encodes for a member of the matrix metalloproteinase (MMP) protein family involved in the breakdown of extracellular matrix in several processes. MMP8 functions in the degradation of type I, II and III collagens. Described as upregulated as part of a 7-gene predictive signature of early primary prefibrotic myelofibrosis (prePMF) [18]. Upregulated in PB from patients with PMF and related neoplasms [20] and directly involved in HSC mobilization and trafficking [21].
***PBXIP1***	PBX homeobox interacting protein 1	Encodes for a protein that inhibits the transcriptional activation potential of PBX1 homeodomain protein (PBX1) by preventing its binding to DNA. This protein can also interact with estrogen receptors alpha and beta, promoting the proliferation of some cancers. PBX1 regulates the equilibrium between self-renewal and differentiation of HSCs and the stem cell transcriptional program directed by this protein drives tumor progression in MPNs [26].
***PTGS1***	Prostaglandin-endoperoxide synthase 1	This enzyme catalyzes the conversion of arachidonate to prostaglandin with a dual function as both a cyclooxygenase and as a peroxidase, regulates angiogenesis in endothelial cells and may promote cell proliferation during tumor progression. It is involved in the biosynthesis pathway of prostanoids, in particular in the stomach and platelets. PTGS1 overexpression has been previously implicated in MPNs [27] and mutations in this gene are associated with bleeding and platelet disfunction [28].

**Table 2:** Functions and relationship with MPNs of the potential biomarkers of interest exclusively for ET patients with *CALR* mutations

Gene	Protein	Function and relationship with MPNs
***BMP6***	Bone morphogenetic protein 6	Encodes a secreted ligand of the TGF-β (transforming growth factor-beta) superfamily of proteins that plays essential roles in many developmental processes including cartilage and bone formation. BMP6 is also an endogenous regulator of iron levels [29] that binds various TGF-β receptors triggering the recruitment and activation of SMAD transcription factors that regulate gene expression. Bone morphogenetic proteins are overexpressed in PMF having a role in the aberrant BM matrix homeostasis in these patients [30]. These proteins have been also described to regulate differentiation of human promyelocytic leukemia cells [31] and BMP6 promoter methylation is likely to be a common epigenetic event at later stages of adult T-cell leukemia [32].
***CEACAM8***	Carcinoembryonic antigen-related cell adhesion molecule 8	Encodes a cell surface glycoprotein that plays a role in cell adhesion in a calcium-independent manner. Protein overexpressed in PMF and implicated in key processes involved in the pathogenesis of this disease, such as cell adhesion, cellular invasiveness, angiogenesis and inflammation [33].
***ITK***	IL2 inducible T cell kinase	Encodes an intracellular tyrosine kinase expressed in T-cells. It plays an essential role in regulation of the adaptive immune response and in T-cell growth, signaling and function. Common target in T-cell diseases [37] but has not been previously linked to MPNs.
***LCN2***	Lipocalin 2	Encodes a protein of the lipocalin family of proteins that transport small hydrophobic molecules such as lipids, steroid hormones, and retinoids. LCN2 is a neutrophil gelatinase-associated lipocalin that plays a role in innate immunity and seems to be involved in multiple cellular processes, including maintenance of skin homeostasis, and suppression of invasiveness and metastasis. LCN2 is a proinflammatory mediator that contributes to MPNs initiation and progression [34]. It has been found elevated in the plasma of MPN patients [35] and cells with the p.V617F mutation cause DNA damage to adjacent normal cells through the secretion of this molecule [36].
***MAN1A1***	Mannosidase alpha class 1 A member 1	Encodes a class I mammalian Golgi 1,2-mannosidase, a type II transmembrane protein. This protein catalyzes the hydrolysis of three terminal mannose residues from peptide-bound Man(9)-GlcNAc(2) oligosaccharides and it is involved in the asparagine N-linked glycosylation. The reduced expression of this gene in some breast cancers leads to aberrant N-glycosylation and impaired survival [38] and is downregulated in the chronic phase of chronic myeloid leukemia (CML) [39].
***MME***	Membrane metalloendopeptidase (also known as CD10)	Encodes a type II transmembrane glycoprotein present on leukemic cells of pre-B phenotype but also in normal tissues with an endopeptidase activity that cleaves peptides on the amino side of hydrophobic residues and inactivates several peptide hormones. Important cell surface marker in the diagnosis of human acute lymphocytic leukemia (ALL) and found downregulated in PV and PMF granulocytes but not in ET [40].
***PRG2***	Proteoglycan 2	Encodes a pro-eosinophil major basic protein, an eosinophilic marker. Until date it has not been directly associated with MPNs.

**Table 3:** Functions and relationship with MPNs of the potential biomarkers of interest exclusively for ET patients with *JAK2* mutations

Gene	Protein	Function and relationship with MPNs
***ARG1***	Arginase 1	Encodes a protein that catalyzes the hydrolysis of arginine to ornithine and urea. Arginine metabolism is a critical regulator of innate and adaptive immune responses. The myeloid-derived suppressor cells of MPN patients show an increased expression of this protein leading to arginine depletion and suppressor T cell activity [41].
***CAST***	Calpastatin	Encodes a calpain inhibitor. The calpain/calpastatin system is involved in numerous membrane fusion events, including platelet and erythrocyte aggregation. This gene has not been directly associated with MPNs but decreased calpastatin expression and elevated calpain activity has been observed in acute myeloid leukemia [42].
***CD177***	CD177	Encodes a glycosyl-phosphatidylinositol (GPI)-linked cell surface glycoprotein with a role in neutrophil activation. It can bind platelet endothelial cell adhesion molecule-1 and function in neutrophil transmigration. The overexpression of this gene was proposed as a marker of abnormal neutrophil production in patients with MPNs [43].
***CLEC5A***	C-type lectin domain containing 5 A	Encodes a member of the C-type lectin/ C-type lectin-like domain (CTL/CTLD) superfamily of proteins critical for myeloid differentiation. Its dysregulation has been associated with myelodysplastic syndromes [25].
***DAPP1***	Dual adaptor of phosphotyrosine and 3-phosphoinositides 1	Encodes a protein located in plasma membrane predicted to be involved in signal transduction enabling phosphatidylinositol-3,4,5-trisphosphate binding activity and phosphatidylinositol-3,4-bisphosphate binding activity. Positive regulator of tyrosine kinase-induced cell responses [44] but not related to MPNs until date.
***EPS15***	Epidermal growth factor receptor pathway substrate 15	Encodes a protein that is part of the EGFR pathway and present at clathrin-coated pits. It is involved in receptor-mediated endocytosis of EGF and may be involved in the regulation of mitogenic signals and control of cell proliferation. Its fusion with *KMT2A* has been observed in patients with acute myeloblastic leukemia [45].
***IGHM***	Immunoglobulin heavy constant mu	Encodes the C-region of the immunoglobulin heavy chain mu, which defines the IgM isotype. Membrane-bound IgM induces the phosphorylation of CD79A and CD79B by the Src family of protein tyrosine kinases and it may cause death of cells by apoptosis. Not previously associated with MPNs.
***IL18RAP***	Interleukin 18 receptor accessory protein	Encodes an accessory subunit of the heterodimeric receptor for interleukin 18, a proinflammatory cytokine involved in inducing cell-mediated immunity. It enhances the IL18-binding activity of the IL18 receptor, so it is involved in IL18-dependent signal transduction, leading to NFκB and JNK activation. Not previously associated with MPNs.
***OLFM4***	Olfactomedin 4	Encodes a member of the olfactomedin family that is an extracellular matrix glycoprotein that facilitates cell adhesion and promotes tumor growth. Previously associated with myelofibrosis transformation of ET patients [46] and seems to be a survival factor for primitive leukemia cells [47].
***OLR1***	Oxidized low density lipoprotein receptor 1	Encodes a low-density lipoprotein receptor that belongs to the C-type lectin superfamily. It internalizes and degrades oxidized low-density lipoprotein and may be involved in the regulation of Fas-induced apoptosis. This gene is upregulated in patients with chronic myelomonocytic leukemia [48].
***RIOK3***	RIO kinase 3	Encodes a member of the right open reading frame (RIO) kinase gene family, a serine/threonine kinase with a role in the processing of the pre-40 S ribosomal subunit, 21 S pre-rRNA and in NFκB signaling pathway inhibiting CASP10 isoform 7-mediated activation. Not previously associated with MPNs.
***SELP***	Selectin P	Encodes a 140 kDa protein stored in the alpha-granules of platelets that is involved in platelet activation and degranulation, as well as in the interaction of platelets with leukocytes. Vascular endothelial expression of the p.V617F mutation in JAK2 has been described to increase the expression of SELP promoting a pro-thrombotic state [49].
***THBS1***	Thrombospondin 1	Encodes a subunit of a disulfide-linked homotrimeric protein, an adhesive glycoprotein that mediates cell-to-cell and cell-to-matrix interactions. This protein can bind to fibrinogen, fibronectin, laminin, type V collagen and integrins alpha-V/beta-1 with roles in platelet aggregation, angiogenesis, and tumorigenesis. *THBS1* is overexpressed in PV and ET patients [50] and PMF megakaryocytes secrete this protein leading to angiogenesis and myelofibrosis development [51].

## Discussion

The knowledge of the molecular pathogenesis of chronic MPNs has undergone considerable progress in recent decades, especially after the description of the presence of a single nucleotide mutation in *JAK2* (p.V617F) in the vast majority of patients with these diseases in 2005. Subsequently, *MPL* mutations were found in a small proportion of patients and, finally, it was possible to determine that the third major mutated gene was *CALR* in 2013 thanks to the application of massive sequencing technologies. Taken together, these aberrations activate cell signaling primarily through the JAK2/STAT pathway, justifying the relationship between these diseases, but also through other related signaling pathways that lead to cell proliferation.

Apart from unveiling possible therapeutic targets and a better understanding of the mechanisms that trigger these diseases, the description of these alterations represented a considerable diagnostic revolution since it allowed the clonal nature of the disease to be demonstrated in a simple way. However, there are still several issues that have not been fully resolved, such as the fact that the same alterations can lead to different, although related, clinical phenotypes. Furthermore, in the case of ET patients no targeted therapies have been approved and there is still a certain percentage of patients with this disease that do not show any of the genetic alterations mentioned.

The present work aims to re-analyze the gene expression profiles deposited in public databases of patients with ET with *JAK2* and *CALR* mutations and healthy donors obtained from both CD34 + cells and mature neutrophils. The study of the transcriptional alterations conserved between undifferentiated CD34 + cells and mature myeloid cells might help to discover molecular players of special importance for ET patients, pointing to new potential therapeutic targets.

The GSE103237 dataset included CD34 + cell transcriptomic data from BM obtained from 24 ET patients (7 *CALR*-mutated and 17 *JAK2*-mutated) and 15 healthy donors [8]. In the original study, the authors focused on the differences in the expression profile between *CALR*-mutated and *JAK2*-mutated CD34 + cells that supported the existence of different clinical entities for patients with both mutations. Thus, they showed that the expression of several genes involved in DNA repair, chromatin remodeling, splicing, chromatid cohesion and several genes involved in thrombin signaling and platelet activation were downregulated, which could explain the lower risk of thrombosis associated with *CALR* mutations.

On the other hand, the GSE54644 set contained expression data from PB neutrophils obtained from 39 ET patients (14 *CALR*-mutated and 25 *JAK2*-mutated) and 11 healthy donors [7]. In this study the authors showed that a transcriptional signature consistent with activated JAK2 signaling was present in all MPN patients regardless of clinical phenotype or mutational status. These results pointed to a shared mechanism of transformation by *JAK2* and *CALR* mutations, demonstrating the central importance of the JAK2/STAT pathway in MPN pathogenesis. Indeed, the mutant CALR was subsequently shown to drive signaling by activating JAK2 through the thrombopoietin receptor MPL [52–54].

General re-analysis of both datasets showed differences and similarities between CD34 + cells and mature neutrophils from ET patients.

With respect to the differences, more DEGs were found in CD34 + samples than in mature neutrophils. In fact, immature BM CD34 + cells show more exclusive altered hallmarks (17 in *CALR*-mutated samples and 16 in *JAK2*-mutated patients) participating in a wide variety of biological processes. Some of these processes were common to *JAK2* and *CALR*-mutated patients (allograft rejection, angiogenesis, apical surface, reactive oxygen species pathway, hedgehog signaling, pancreas beta cells, fatty acid metabolism, and peroxisome).

Focusing on the similarities, BM CD34 + cells and PB neutrophils shared many of the altered hallmarks regardless of mutation type. In fact, all but one of the hallmarks altered in neutrophils (mitotic spindle in *CALR*-mutated patients) were also found to be altered in immature CD34 + cells. Interestingly, the majority of these hallmarks were common to *JAK2* and *CALR*-mutated patients, providing evidence about shared mechanisms of interest for both types of patients apart for the already described JAK2/STAT activation [7] and not only in mature neutrophils but also in immature CD34 + cells. Likewise, the differences between patients harboring *JAK2* and *CALR* mutations support previous analyses in PB [8] but also translate these results to BM.

In addition to the hallmarks, we particularly focused on similarly expressed genes in CD34 + cells and mature neutrophils since they might be relevant molecular players in ET pathogenesis that could be targeted. In this sense, we could only observe the conservation of the expression of 338 and 919 genes between the PB neutrophils and BM CD34 + cells of *CALR* and *JAK2*-mutated patients, respectively. Among them, we have obtained an expression signature of six genes common to patients with mutations in *CALR* and *JAK2* (*BPI*, *CRISP3, LTF*, *MMP8*, and *PTGS1* upregulated, and *PBXIP1* downregulated), and two other expression signatures of seven (*BMP6, CEACAM8, ITK, LCN2*, and *PRG2* upregulated, and *MAN1A1* and *MME* downregulated) and 13 genes (*ARG1, CAST, CD177, CLEC5A, DAPP1, EPS15, IL18RAP, OLFM4, OLR1, RIOK3, SELP*, and *THBS1* upregulated, and *IGHM* downregulated) exclusive to patients with ET harboring mutations in *CALR* and *JAK2*, respectively. The expression of many of these genes was confirmed in PBMCs RNA-seq data from an independent cohort of ET patients, although the small number of patients and the heterogeneity among them resulted in non-significant results in many cases. Additionally, there might be some differences compared to the results obtained from PB neutrophil samples due to the analysis of different types of mature blood cells. The selected genes that were common to *CALR* and *JAK2*-mutated patients were involved in very specific biological processes that could be relevant to the pathogenesis of the disease, so it would be worthwhile to carry out further studies on these processes in both types of patients. For example, *MAN1A1*, one of the genes exclusively downregulated in *CALR*-mutated patients is involved in N-linked glycosylation. In support of our results, it has recently been described that genes in the N-glycosylation pathway are differentially depleted in mutant *CALR*-transformed cells. In these cells, chemical inhibition of N-glycosylation impaired their growth [55]. In addition, it is important to note that most of these genes have been previously related to MPNs or hematologic cancers.

Globally, the methodology used in this study consists of a detailed bioinformatic analysis that takes advantage of previously published data to observe them from a new perspective and has allowed the identification of some features and genes that may be relevant in the pathogenesis of ET in patients with *CALR* or *JAK2* mutations. Strict criteria have been employed in the analysis of these data to highlight only relevant processes and genes, and the expression values of many of these highlighted genes have been validated in an independent cohort of patients using RNA-seq data. However, this study also has some limitations. Firstly, the data available for each patient are different in each of the databases, which has made it difficult to associate the results obtained with specific *CALR* or *JAK2* mutations. Moreover, the low number and heterogeneity of the patients included in the RNA-seq dataset has not made it possible to confirm all the results obtained in the analysis of the arrays. Finally, there is no expression data for the intermediate cells between the BM CD34 + stem cells and the PB neutrophils or PBMCs, as the only available datasets in GEO that include transcriptomic data from ET patients with *CALR* or *JAK2* mutations are those used in this study. This limitation, combined with the fact that the analyzed samples from each cell type are derived from different patients, makes it impossible to analyze the dynamics of changes in the clonogenicity of malignant cells in ET.

## Conclusion

The comparison of the transcriptomic profile of bone marrow (BM) CD34 + cells and peripheral blood (PB) neutrophils from ET patients and healthy donors highlights molecular similarities and differences according to the degree of maturation of the malignant clone and the type of mutation, which enables delving into the molecular pathogenic mechanisms. Although our results show that most of the altered hallmarks in neutrophils were also found in CD34 + cells in both *CALR* and *JAK2*-mutated patients, only a few genes showed a similar expression pattern in both types of cells. These genes are of special interest since they may reveal altered sustained biological processes throughout the entire malignant hematopoietic lineage and might be relevant to the development or progression of the disease. Thus, the progression of the disease to MF or the severity of symptoms presented in ET patients could ultimately depend on these genes and processes and hence, they could be studied as future therapeutic targets for ET patients according to their driver mutation.

### Electronic supplementary material

Below is the link to the electronic supplementary material.

Supplementary Material 1: Table S1. Biological and clinical characteristics of the patients included in the study.

Supplementary Material 2: Table S2. Results of microarray analysis when comparing *CALR*-mutated ET patients vs. healthy donors. Non-differentially expressed genes are shaded in red (adjusted *p*-value > 0.01).

Supplementary Material 3: Table S3. Results of microarray analysis when comparing *JAK2*-mutated ET patients vs. healthy donors. Non-differentially expressed genes are shaded in red (adjusted *p*-value > 0.01).

Supplementary Material 4: Table S4. Equally expressed genes in the BM and PB of *CALR*-mutated ET patients.

Supplementary Material 5: Table S5. Equally expressed genes in the BM and PB of *JAK2*-mutated ET patients.

Supplementary Material 6: Supplementary Figure 1. Results of RNA-seq analysis of samples from PBMCs for the gene signature found in this study when comparing (**a**) *CALR*-mutated or (**b**) *JAK2*-mutated ET patients vs. healthy donors. Individual transcript per million (TPM) values and means are represented.

Supplementary Material 7: Supplementary Figure 2. Tables showing data from RNA-seq analysis of samples from PBMCs for the gene signature found in this study when comparing (**a**) *CALR*-mutated or (**b**) *JAK2*-mutated ET patients vs. healthy donors. Mean log2FC values and adjusted *p-*values are represented.

## Data Availability

The datasets used during the current study are available in the GEO repository under accession numbers GSE103237, GSE54644, and GSE156336.

## Abbreviations

ET: essential thrombocythemia
HSC: hematopoietic stem cell
BM: bone marrow
JAK2: Janus kinase 2
CALR: calreticulin
MPL: thrombopoietin receptor
PB: peripheral blood
MPNs: myeloproliferative neoplasms
PV: polycythemia vera
PMF: primary myelofibrosis
GEO: Gene Expression Omnibus
PBMCs: peripheral blood mononuclear cells
DEGs: differentially expressed genes
GSEA: Gene Set Enrichment Analysis

## References

[1] Guijarro-Hernández A, Vizmanos JL (2021). A broad overview of signaling in Ph-Negative Classic Myeloproliferative Neoplasms. Cancers.

[2] Mead AJ, Mullally A (2017). Myeloproliferative neoplasm stem cells. Blood.

[3] Tefferi A, Pardanani A (2019). Essential thrombocythemia. N Engl J Med.

[4] Szuber N, Mudireddy M, Nicolosi M, Penna D, Vallapureddy RR, Lasho TL et al. 3023 Mayo Clinic Patients With Myeloproliferative Neoplasms: Risk-Stratified Comparison of Survival and Outcomes Data Among Disease Subgroups. *Mayo Clin Proc*. 2019;94:599–610. 10.1016/j.mayocp.2018.08.022. 10.1016/j.mayocp.2018.08.022 30824279

[5] Li B, Rampal RK, Xiao Z (2019). Targeted therapies for myeloproliferative neoplasms. Biomark Res.

[6] Harrison CN, Mead AJ, Panchal A, Fox S, Yap C, Gbandi E (2017). Ruxolitinib vs best available therapy for ET intolerant or resistant to hydroxycarbamide. Blood.

[7] Rampal R, Al-Shahrour F, Abdel-Wahab O, Patel JP, Brunel JP, Mermel CH (2014). Integrated genomic analysis illustrates the central role of JAK-STAT pathway activation in myeloproliferative neoplasm pathogenesis. Blood.

[8] Zini R, Guglielmelli P, Pietra D, Rumi E, Rossi C, Rontauroli S (2017). CALR mutational status identifies different disease subtypes of essential thrombocythemia showing distinct expression profiles. Blood Cancer J.

[9] Wu T, Hu E, Xu S, Chen M, Guo P, Dai Z (2021). clusterProfiler 4.0: a universal enrichment tool for interpreting omics data. Innov (Camb).

[10] Dolgalev I. Msigdbr: MSigDB gene sets for multiple organisms in a tidy data format. R package version 7.5.1. 2022. https://igordot.github.io/msigdbr/.

[11] Lennartsson A, Pieters K, Ullmark T, Vidovic K, Gullberg U (2003). AML-1, PU.1, and Sp3 regulate expression of human bactericidal/permeability-increasing protein. Biochem Biophys Res Commun.

[12] Yokota A, Huo L, Lan F, Wu J, Huang G (2020). The clinical, molecular, and mechanistic basis of RUNX1 mutations identified in hematological malignancies. Mol Cells.

[13] Sakurai H, Harada Y, Ogata Y, Kagiyama Y, Shingai N, Doki N (2017). Overexpression of RUNX1 short isoform has an important role in the development of myelodysplastic/myeloproliferative neoplasms. Blood Adv.

[14] Guglielmelli P, Bartalucci N, Contini E, Rotunno G, Pacilli A, Romagnoli S (2019). Involvement of RUNX1 pathway is a common event in the leukemic transformation of chronic myeloproliferative neoplasms (MPNs). Blood.

[15] Irino T, Uemura M, Yamane H, Umemura S, Utsumi T, Kakazu N (2011). JAK2 V617F-dependent upregulation of PU.1 expression in the peripheral blood of myeloproliferative neoplasm patients. PLoS ONE.

[16] Van Loo PF, Bouwman P, Ling KW, Middendorp S, Suske G, Grosveld F (2003). Impaired hematopoiesis in mice lacking the transcription factor Sp3. Blood.

[17] Schardt JA, Keller M, Seipel K, Pabst T (2015). Functional interplay of SP family members and nuclear factor Y is essential for transcriptional activation of the human calreticulin gene. Biochim Biophys Acta.

[18] Skov V, Burton M, Thomassen M, Stauffer Larsen T, Riley CH, Brinch Madelung A (2016). A 7-gene signature depicts the biochemical profile of early prefibrotic myelofibrosis. PLoS ONE.

[19] Leng D, Miao R, Huang X, Wang Y (2018). In silico analysis identifies CRISP3 as a potential peripheral blood biomarker for multiple myeloma: from data modeling to validation with RT-PCR. Oncol Lett.

[20] Skov V, Thomassen M, Kjær L, Riley C, Stauffer Larsen T, Bjerrum OW (2018). Extracellular matrix-related genes are deregulated in peripheral blood from patients with myelofibrosis and related neoplasms. Blood.

[21] Steinl C, Essl M, Schreiber TD, Geiger K, Prokop L, Stevanović S (2013). Release of matrix metalloproteinase-8 during physiological trafficking and induced mobilization of human hematopoietic stem cells. Stem Cells Dev.

[22] Yamada Y, Amagasaki T, Jacobsen DW, Green R (1987). Lactoferrin binding by leukemia cell lines. Blood.

[23] Butler TW, Heck LW, Huster WJ, Grossi CE, Barton JC (1988). Assessment of total immunoreactive lactoferrin in hematopoietic cells using flow cytometry. J Immunol Methods.

[24] Brown RD, Rickard KA, Kronenberg H (1985). Lactoferrin in the myeloproliferative disorders: a search for granulopoietic regulator defects. Br J Haematol.

[25] Müller CI, Luong QT, Shih LY, Jones LC, Desmond JC, Kawamata N (2008). Identification of marker genes including RUNX3 (AML2) that discriminate between different myeloproliferative neoplasms and normal individuals. Leukemia.

[26] Muggeo S, Crisafulli L, Uva P, Fontana E, Ubezio M, Morenghi E (2021). PBX1-directed stem cell transcriptional program drives tumor progression in myeloproliferative neoplasm. Stem Cell Reports.

[27] Hasselbalch HC, Thomassen M, Riley CH, Kjær L, Larsen TS, Jensen MK (2014). Whole blood transcriptional profiling reveals deregulation of oxidative and antioxidative defence genes in myelofibrosis and related neoplasms. Potential implications of downregulation of Nrf2 for genomic instability and disease progression. PLoS ONE.

[28] Palma-Barqueros V, Bohdan N, Revilla N, Vicente V, Bastida JM, Rivera J (2021). PTGS1 gene variations associated with bleeding and platelet dysfunction. Platelets.

[29] Camaschella C (2009). BMP6 orchestrates iron metabolism. Nat Genet.

[30] Bock O, Höftmann J, Theophile K, Hussein K, Wiese B, Schlué J (2008). Bone morphogenetic proteins are overexpressed in the bone marrow of primary myelofibrosis and are apparently induced by fibrogenic cytokines. Am J Pathol.

[31] Topić I, Ikić M, Ivčević S, Kovačić N, Marušić A, Kušec R (2013). Bone morphogenetic proteins regulate differentiation of human promyelocytic leukemia cells. Leuk Res.

[32] Taniguchi A, Nemoto Y, Yokoyama A, Kotani N, Imai S, Shuin T (2008). Promoter methylation of the bone morphogenetic protein-6 gene in association with adult T-cell leukemia. Int J Cancer.

[33] Hasselbalch HC, Skov V, Larsen TS, Thomassen M, Riley CH, Jensen MK (2011). High expression of carcinoembryonic antigen-related cell adhesion molecule (CEACAM) 6 and 8 in primary myelofibrosis. Leuk Res.

[34] Tillmann S, Olschok K, Schröder SK, Bütow M, Baumeister J, Kalmer M (2021). The unfolded protein response is a major driver of LCN2 expression in BCR-ABL- and JAK2V617F-positive MPN. Cancers.

[35] Lu M, Xia L, Liu YC, Hochman T, Bizzari L, Aruch D (2015). Lipocalin produced by myelofibrosis cells affects the fate of both hematopoietic and marrow microenvironmental cells. Blood.

[36] Kagoya Y, Yoshimi A, Tsuruta-Kishino T, Arai S, Satoh T, Akira S (2014). JAK2V617F + myeloproliferative neoplasm clones evoke paracrine DNA damage to adjacent normal cells through secretion of lipocalin-2. Blood.

[37] Zhong Y, Johnson AJ, Byrd JC, Dubovsky JA (2014). Targeting Interleukin-2-Inducible T-cell kinase (ITK) in T-Cell related Diseases. Postdoc J.

[38] Legler K, Rosprim R, Karius T, Eylmann K, Rossberg M, Wirtz RM (2018). Reduced mannosidase MAN1A1 expression leads to aberrant N-glycosylation and impaired survival in breast cancer. Br J Cancer.

[39] Spivak JL, Considine M, Williams DM, Talbot CC, Rogers O, Moliterno AR (2014). Two clinical phenotypes in polycythemia vera. N Engl J Med.

[40] Wang JC, Kundra A, Andrei M, Baptiste S, Chen C, Wong C (2016). Myeloid-derived suppressor cells in patients with myeloproliferative neoplasm. Leuk Res.

[41] Niapour M, Farr C, Minden M, Berger SA (2012). Elevated calpain activity in acute myelogenous leukemia correlates with decreased calpastatin expression. Blood Cancer J.

[42] Passamonti F, Pietra D, Malabarba L, Rumi E, Della Porta MG, Malcovati L (2004). Clinical significance of neutrophil CD177 mRNA expression in Ph-negative chronic myeloproliferative disorders. Br J Haematol.

[43] Inoue D, Kitaura J, Togami K, Nishimura K, Enomoto Y, Uchida T (2013). Myelodysplastic syndromes are induced by histone methylation–altering ASXL1 mutations. J Clin Invest.

[44] Naudin C, Chevalier C, Roche S (2016). The role of small adaptor proteins in the control of oncogenic signaling driven by tyrosine kinases in human cancer. Oncotarget.

[45] De Braekeleer E, Meyer C, Douet-Guilbert N, Basinko A, Le Bris MJ, Morel F (2011). Identification of MLL partner genes in 27 patients with acute leukemia from a single cytogenetic laboratory. Mol Oncol.

[46] Hasselbalch HC, Skov V, Stauffer Larsen T, Thomassen M, Hasselbalch Riley C, Jensen MK (2014). Transcriptional profiling of whole blood identifies a unique 5-gene signature for myelofibrosis and imminent myelofibrosis transformation. PLoS ONE.

[47] Suknuntha K, Ishii Y, Hu K, Brayan M, Yang DT, Swanson S (2014). Induced pluripotent stem cell model of chronic myeloid leukemia revealed Olfactomedin 4 as a novel survival factor for primitive leukemia cells. Blood.

[48] Franzini A, Pomicter AD, Yan D, Khorashad JS, Tantravahi SK, Than H (2019). The transcriptome of CMML monocytes is highly inflammatory and reflects leukemia-specific and age-related alterations. Blood Adv.

[49] Guy A, Gourdou-Latyszenok V, Le Lay N, Peghaire C, Kilani B, Dias JV (2019). Vascular endothelial cell expression of JAK2^V617F^ is sufficient to promote a pro-thrombotic state due to increased P-selectin expression. Haematologica.

[50] Gangaraju R, Song J, Kim SJ, Tashi T, Reeves BN, Sundar KM (2020). Thrombotic, inflammatory, and HIF-regulated genes and thrombosis risk in polycythemia vera and essential thrombocythemia. Blood Adv.

[51] Mondet J, Hussein K, Mossuz P. Circulating cytokine levels as markers of inflammation in Philadelphia negative myeloproliferative neoplasms: diagnostic and prognostic interest. Mediators Inflamm. 2015;670580. 10.1155/2015/670580. 10.1155/2015/670580 PMC4617441 26525644

[52] Araki M, Yang Y, Masubuchi N, Hironaka Y, Takei H, Morishita S (2016). Activation of the thrombopoietin receptor by mutant calreticulin in CALR-mutant myeloproliferative neoplasms. Blood.

[53] Chachoua I, Pecquet C, El-Khoury M, Nivarthi H, Albu RI, Marty C (2016). Thrombopoietin receptor activation by myeloproliferative neoplasm associated calreticulin mutants. Blood.

[54] Elf S, Abdelfattah NS, Chen E, Perales-Patón J, Rosen EA, Ko A (2016). Mutant calreticulin requires both its mutant C-terminus and the thrombopoietin receptor for oncogenic transformation. Cancer Discov.

[55] Jutzi JS, Marneth AE, Ciboddo M, Guerra-Moreno A, Jiménez-Santos MJ, Kosmidou A (2022). Whole-genome CRISPR screening identifies N-glycosylation as a genetic and therapeutic vulnerability in *CALR*-mutant MPN. Blood.

