# Supplementary figures and images for: Transcriptomic comparison of bone marrow CD34 + cells and peripheral blood neutrophils from ET patients with JAK2 or CALR mutations
Source: BMC Genom Data. 2023 Aug 7;24:40. doi: 10.1186/s12863-023-01142-5 (PMC10408115; doi:10.1186/s12863-023-01142-5)

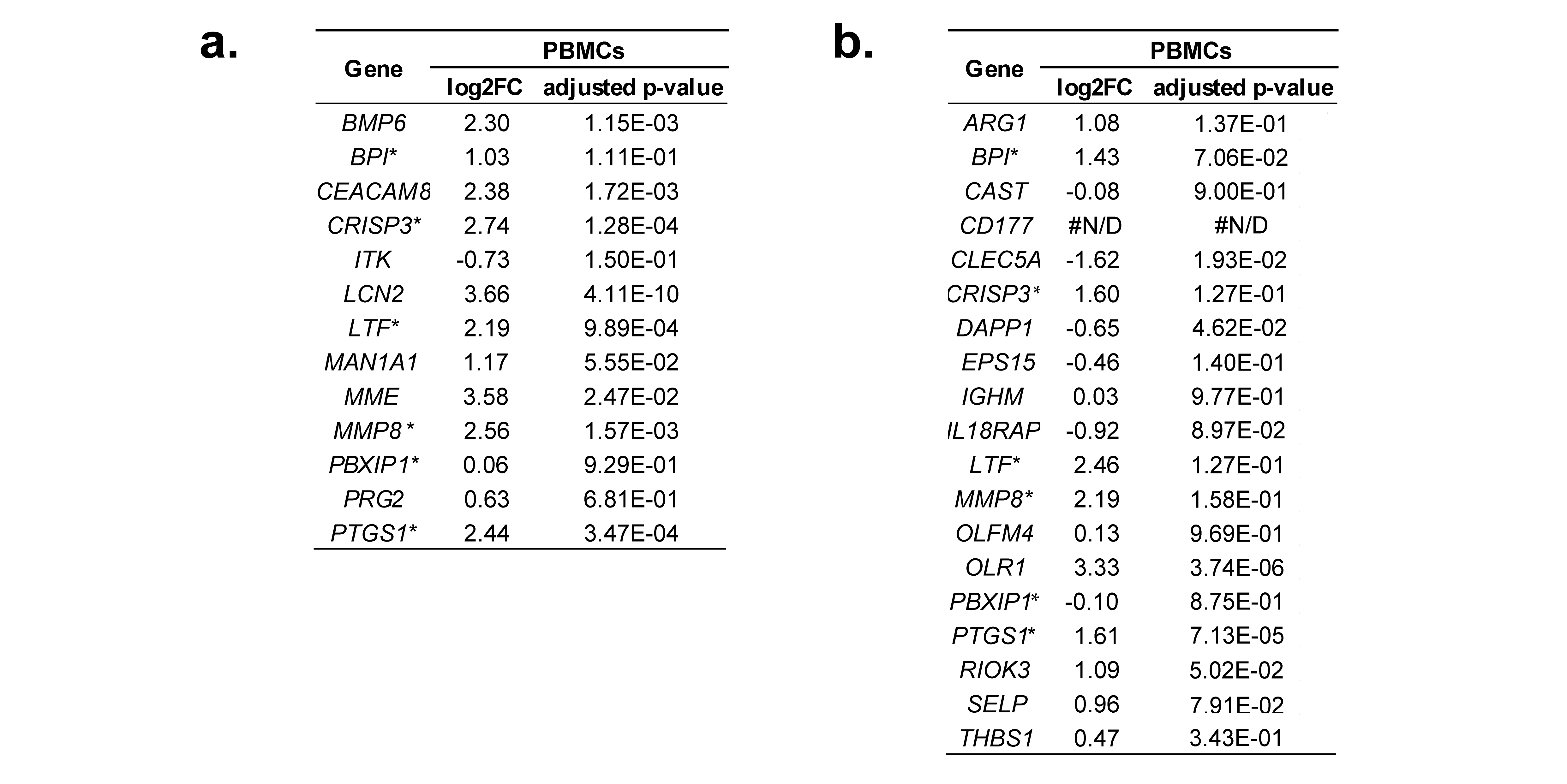

Supplement: Supplementary file 7 — Supplementary Material 7: Supplementary Figure 2. Tables showing data from RNA-seq analysis of samples from PBMCs for the gene signature found in this study when comparing (a) CALR-mutated or (b) JAK2-mutated ET patients vs. healthy donors. Mean log2FC values and adjusted p-values are represented. [file 12863_2023_1142_MOESM7_ESM.tif]
